# Incidence for volar locking plate removal following distal radius fracture surgery

**DOI:** 10.1007/s00402-020-03565-6

**Published:** 2020-08-30

**Authors:** Vili Palola, Ville Ponkilainen, Tuomas Huttunen, Antti Launonen, Ville M. Mattila

**Affiliations:** 1 Faculty of Medicine and Health Technology, Tampere University, 33520 Tampere, Finland; 2 Department of Orthopaedics and Traumatology, Tampere University Hospital, Tampere, Finland; 3 Faculty of Medicine and Health Technology, Tampere University, Tampere University Hospital, Tampere, Finland; 4 Department of Emergency, Anesthesia and Pain Medicine, Tampere University Hospital, Tampere, Finland; 5 The Division of Orthopedics and Biotechnology, Department of Clinical Science, Intervention and Technology (CLINTEC), Karolinska Institutet, Stockholm, Sweden; 6 COXA Hospital for Joint Replacement, Biokatu 6, 33520 Tampere, Finland

**Keywords:** Distal radius fracture, Surgery, Volar plate, Plate removal, Epidemiology

## Abstract

**Introduction:**

Distal radius fracture is the most common fracture in adults. The most common treatment for distal radius fracture is non-operative cast immobilization, although there are injuries that require surgical treatment. During the past decade, studies have reported a large increase in the surgical treatment of distal radius fractures with open reduction and internal fixation using volar locking plates. The aim of this study was to investigate the incidence and trends for plate removal after plate fixation of distal radius fractures.

**Materials and methods:**

The study covered all patients 18 years of age and older who had a surgically treated distal radius fracture with open reduction and internal fixation in Finland between 1998 and 2016. Patient data were obtained from the Finnish National Hospital Discharge Register. The association between increased number of platings and plate removals was examined by calculating the removal rates. The study population comprises all patients on a national level, and therefore we did not use statistical testing to analyze the data.

**Results:**

A total of 18,298 patients had surgically treated distal radius fracture with volar plate in Finland during the 19-year study period from January 1, 1998 to December 31, 2016. The number of plate removal operations over the same time period was 2560. The removal rates decreased from over 20% in 1998 to less than 12% in 2016. The mean time period between plating and plate removal operations was 367 days. Most of the plate removals (*n* = 2235; 87.3%) were conducted during the first 2 years after plating.

**Conclusion:**

Plate removals have not increased as rapidly as plating operations. The removal rate has declined markedly during the last decade. Nowadays, approximately 11% of distal radius plates are removed.

## Introduction

Distal radius fracture (DRF) is the most common fracture in adults [1]. Falling from standing height is the most common trauma mechanism for elderly people [1, 2]. Among the adolescent population, the incidence of DRFs is higher in males than in females [3]. However, in the adult population, women have a two to three times greater risk for DRFs than men [1, 4]. In addition, osteoporosis is a major risk factor for DRF in the elderly population. As the active elderly population continues its growth, prevalence of distal radius fractures will most probably continue to rise [5, 6].

The overall distal radius fracture incidence varies between 100 and 350/100,000 person-years among different studies [3, 4, 7–9]. Flinkkilä et al. reported that the incidence of DRF was 363/100,000 person-years in women and 147/100,000 person-years in men, giving a total incidence of 258/100,000 person-years in Finland in 2008 [4].

The treatment of distal radius fractures is a major public health concern, since fall-related DRFs seem to increase in the working population [10, 11]. The most common treatment for distal radius fracture is non-operative cast immobilization, although there are injuries that require surgical treatment [12, 13]. Main indications for surgery are unstable dislocated DRF and comminuted intra-articular fracture, especially in young people, as it has been found that surgical treatment has a positive effect on the functional outcome in short-term follow-ups [14–16]. The most common surgical techniques used for the treatment of DRFs are external fixation, percutaneous pinning and open reduction internal fixation (ORIF) [12]. During the past decade, operative treatment has increased due to the development of new surgical techniques [13, 17–19]. One of the most relevant technical advancements was plate with angle-stable locking screws which is placed on the volar side of the fractured wrist [14]. Volar position of the plate causes less complications and the fixation is more secure [20]. Indeed, several studies have documented a significant increase in surgical treatment, especially ORIF using volar locking plates [13, 17, 18]. Volar lock plating plays a major role in the treatment of distal radius fractures, because nowadays it is the most commonly used surgical technique [21].

Even though volar locking plates have been shown to produce satisfactory results, they sometimes need to be removed [22–24]. To date, there have only been a few published studies on plate removals following the surgical treatment of DRF [23, 25]. One study reported the reasons for plate removals were the following: pain (30%), tenosynovitis (27%), malunion (24%), infection (12%), nonunion (6%) and tendon rupture (3%) [26]. However, while these previous studies report the reasons for plate removal, they suffer from small sample sizes and selected study populations [23, 25, 26].

To the best of our knowledge, no previous studies have investigated the incidence of volar plate removals after DRF at the population level. Hence, the aim of this study was to investigate the nationwide incidence of plate removal after volar lock plating for DRF among the adult population.

## Materials and methods

Patient data for this study were obtained from the Finnish National Hospital Discharge Register (NHDR) between the years 1998 and 2016. The NHDR is an electronic data registry program that is mandatory for all private and public hospitals in Finland. Therefore, the coverage and accuracy of the database are excellent [27]. The NHDR provides data on age, sex, length of hospital stay, domicile of the patient, diagnoses and all treatments done during the hospital stay. All patients have a personal ID number that allows an individual patient to be tracked over the years. Ethical approval was not required for this study because the data did not have identifiable individual participants.

All patients 18 years of age or older who had a distal radius fracture surgically treated with volar locking plate between 1 January 1998 and 31 December 2016 were included. The sample was collected by using the International Classification of Diseases 10th edition (ICD-10) diagnostic codes S52.5 and S52.6 for distal radius fractures and the Nomesco procedural codes NCJ62 and NDJ62 for distal radius surgery. Patients who underwent plate removal after the primary surgery were identified using the operation codes NCU20 and NDU20. In case of multiple operations in the same patient, only the first plating operation was included, and thus each patient existed only once in this data.

The study population comprised all patients on a national level, and therefore we did not use statistical testing to analyze the data. The association between the increased number of platings and plate removals was examined by calculating the removal rates. Ratios for each year were calculated by dividing the plate removals performed during the following 2 years by the number of platings of the current year. We selected a 2-year follow-up period because most of the removals occur during the first 2 years according to the previous studies. Each plated patient was followed for 2 years, and therefore those patients who underwent a plating operation in the last 2 years of the study period (2015 and 2016) were excluded from the results because their follow-up time was less than 2 years.

## Results

**Fig. 1:**
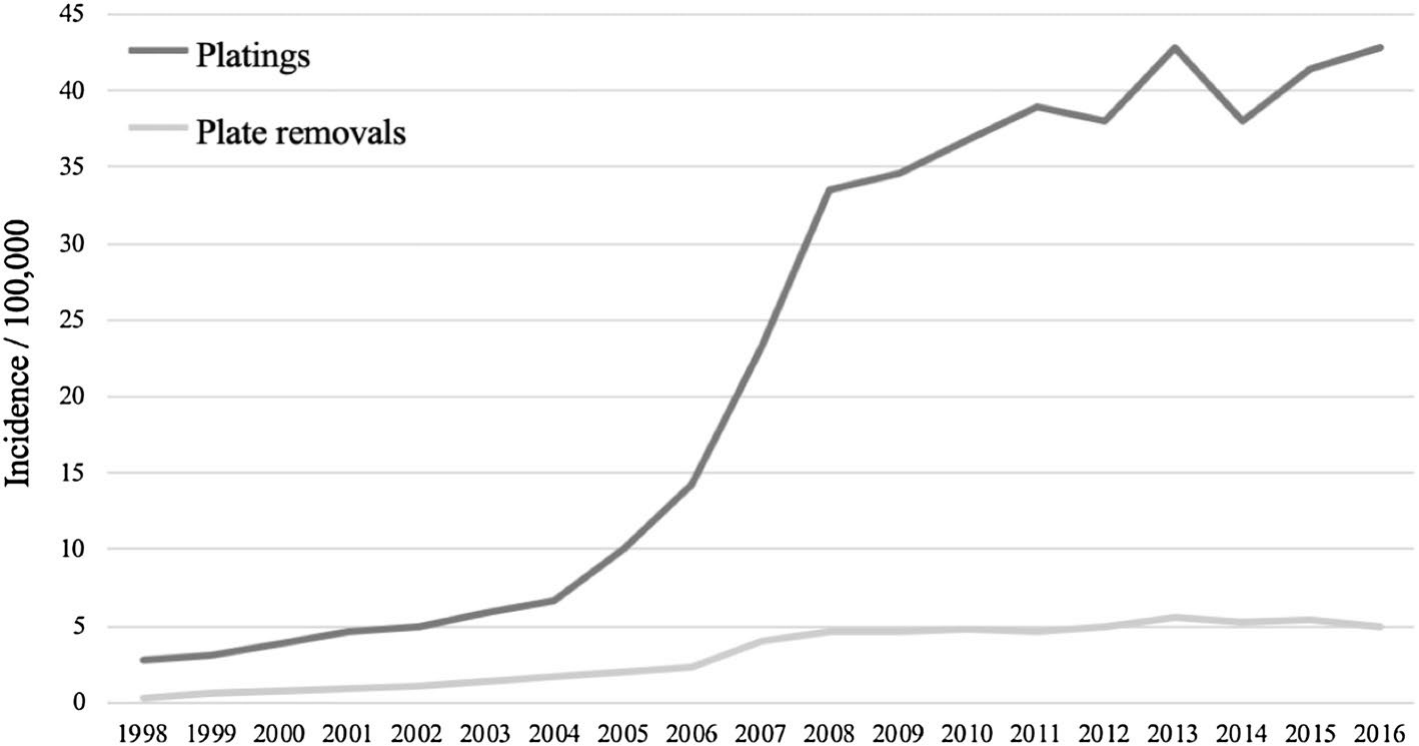
The incidence of plating operations and plate removals in Finland between 1998 and 2016

During the 19-year study period from 1998 to 2016, a total of 18 298 patients had a distal radius fracture surgically treated with volar plate in Finland. The majority of the patients who underwent the plating operation were women (*n* = 12,909; 70.5% women and *n* = 5389; 29.5% men). The mean age at the time of surgery was 60.7 years in women and 48.1 years in men. The number of plating operations increased steadily between 1998 (*n* = 112) and 2004 (*n* = 273) (Fig. 1 and Table 1). After 2004, however, the number of plating operations started to increase notably. Indeed, there was over a fivefold increase in the number of plating operations between 2004 and 2008 (*n* = 1411). The increase in plating incidence slowed down after 2008, however.

**Table 1:** Frequencies and ratios of volar lock plating operations and plate removals by year

Years	Platings	Plate removals^*^ during next 2 years	PR/Plating-ratio (%)
1998	112	29	25.9
1999	121	33	27.3
2000	156	21	13.5
2001	188	39	20.7
2002	202	42	20.8
2003	239	50	20.9
2004	273	67	24.5
2005	420	74	17.6
2006	596	103	17.3
2007	972	191	19.7
2008	1411	186	13.2
2009	1471	183	12.4
2010	1570	198	12.6
2011	1675	195	11.6
2012	1645	166	10.1
2013	1865	209	11.2
2014	1669	191	11.4

*Plate removals were conducted during the following 2 years after the plating

Plate removal operations were conducted for 2560 patients (1634 women, 926 men) between 1998 and 2016. The mean age at the time of plate removal was 52.6 years. The sex-specific mean ages for plate removal were 56.8 years in women and 45.3 years in men. Interestingly, men had a greater removal/plating ratio than women (12.7% in women and 17.2% in men). The number of plate removals doubled between 2006 and 2008, after which the plate removals remained stable (Fig. 1). The mean time period between plating and plate removal operations was 367 days. Most of the plate removals (*n* = 2235; 87.3%) were conducted during the first 2 years after plating.

**Fig. 2:**
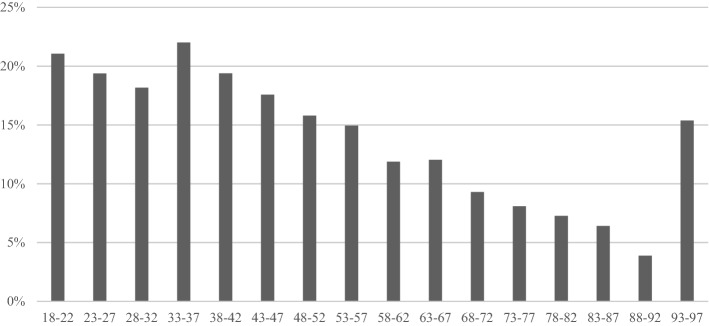
Percentage of plate removal and plating ratio by age group using a 2-year follow-up period after the plating operation

Plate removal operations were conducted within the first 2 years after plating for 29 (25.9%) of the 112 patients who underwent the plating operation in 1998 (Table 1). After that, the removal rate declined steadily to 11% in 2011 and 11.4% in 2014. The removal rate decreased more clearly in 2004 when plating operations started to increase markedly (from 24.5 to 17.6%). Younger patients underwent plate removal more often than older patients (Fig. 2). The removal rate was 18% or more in all age groups aged between 18 and 47 years. After the age of 47, the removal rate started to decline steadily in each age group, and in the 88–92 years age group the rate was only 4%. This decreasing trend is clear when comparing the plate removal rate by age group except for those patients aged 93–97 years who had a 15% removal rate (2/13).

## Discussion

The incidence of distal radius platings with volar locking plate increased markedly between 1998 and 2016. When the incidence of the plating operations started to increase in 2004, removal rates decreased from over 20% in 2004 to approximately 13% in 2008. Interestingly, the number of plate removals did not increase as markedly as platings. However, due to the limitations of our data, we cannot completely explain this result, although it might be related to the design of the volar locking plates themselves which has been gradually improved. Another possible explanation is the advanced skills of surgeons in performing distal radius surgery. These advanced skills lead to better plate positioning and it leads to lower rates of complications and plate removals. Another important finding was the plate removal ratio was higher in younger patients than in older age groups, with the exception of the oldest age group (93–97 years). This interesting finding may be explained by the fact that the requirements of younger persons may be higher and that they are more physically active than the older age groups. The plating of the distal radius can limit the life and activities by impairing grip strength and range of wrist motion of younger persons more easily. Therefore, plate removal operations may be conducted more often for younger patients than for old patients. In addition, young people may request plate removal more often than older people. The high removal rate for the 93–97 years old group is most likely biased because of the low number of platings (2 removals and 13 platings). The higher plate removal ratio seen in men could also be explained in a similar manner.

Our findings of an increased incidence of volar lock plating operations between 2004 and 2008 support previous research [13, 17, 18]. Mattila et al. stated that the factual reason for the increase is not known. Introduction and intense marketing of new plating system, especially plates with locking screws, may play a role in this change [17]. In previous studies, the mean length of plate implantation varies between 330 and 440 days, and thus we selected a 2-year follow-up period because 1-year follow-up would have been too short [23, 26]. In our study, the mean length of implantation was 367 days. A study by Lutsky et al. [25] reported that 37 of 374 plated patients had undergone a plate removal operation, giving a removal ratio of approximately 10%. Their data were collected between 2009 and 2014. When we selected the same time period from our study data (2009–2014), our removal rates varied annually from 10.1 to 12.6%. In another study, Snoddy et al. reported the incidence of plate removals to be between 3 and 4% [26]. Their study included 1 041 patients who had been treated by volar plate. Of these, 33 patients underwent plate removal at their institution between January 2007 and December 2012. The weakness of their study was that they did not contact their patients, and therefore those patients who underwent plate removal elsewhere were missed [25, 26].

Previous studies have reported that pain is the most common reason for plate removal [23, 25, 26]. However, there are no clear criteria as to when plate removal should be conducted, and therefore the surgeon must decide whether plate removal is necessary. There have been several studies that have tried to identify the risk factors for tendon ruptures and tendon irritations after volar plate operation [28, 29]. There are, however, many other reasons, such as infection, tenosynovitis, prominent plate, malunion, nonunion and pain, which may lead to plate removal [23, 25, 26]. In Finland, the DRG price of a plate removal operation after distal radius fracture surgery is 4509.96 euros, yielding an annual nationwide cost of about 900,000 euros. Hence, more research should be done to set clear guidelines for plate removals and to help identify those cases where plate removal is necessary. From an economic point of view, it would not be sensible to remove plates if there is no health benefit.

A key strength of the present study was the accuracy and coverage of the NHDR database, which is collected from all private and public hospitals and other health-care institutions in Finland. Previous studies have reported that the quality of the register is excellent [27]. Other strengths of this study were the 19-year study period and the large nationwide sample size. The weakness of our study was that the NHDR does not contain information on the laterality of the fracture. Therefore, we included only the first volar lock plating operation for each patient, and thus each patient occurs only once in this data. There were a few patients who underwent more than one plating operation during our study period. These patients might have had volar lock plates in both of their hands at the same time, and therefore we cannot be sure which plate was removed. This may have affected the length of plate implantation for these patients. Moreover, the NHDR database does not contain information on the classification of the fracture, nor on indication for plate removal.

## Conclusion

During our study period, the number of volar lock plating operations increased from 112 platings in 1998 to 1 891 platings in 2016. The number of volar lock plate removals did not, however, increase as markedly as plating operations. Between 2006 and 2008, the number of plate removals doubled. Since 2008, however, plate removals have remained steady even though the number of plating operations has slowly increased. Nowadays, the plate removal ratio varies annually between 10 and 12%, which is similar to the findings of previous studies.

## Data Availability

The datasets generated and analysed during the current study are not publicly available due to privacy policy.

## Abbreviations

DRF: Distal radius fracture
ORIF: Open reduction and internal fixation
NHDR: National Hospital Discharge Register
ICD-10: International Classification of Diseases 10th edition
